# Indications for prophylactic osteosynthesis associated with curettage in benign and low-grade malignant primitive bone tumors of the distal femur in adult patients: a case series

**DOI:** 10.1007/s10195-016-0418-7

**Published:** 2016-07-09

**Authors:** Carlo Perisano, Carlo Barone, Daniele Stomeo, Giulio Di Giacomo, Michele Vasso, Alfredo Schiavone Panni, Giulio Maccauro

**Affiliations:** 1 Department of Geriatrics, Neuroscience and Orthopaedics, University Hospital “Agostino Gemelli”, Catholic University of the Sacred Heart School of Medicine, L.go A. Gemelli 1, 00168 Rome, Italy; 2 Department of Internal Medicine, Division of Medical Oncology, University Hospital “Agostino Gemelli”, Catholic University of the Sacred Heart School of Medicine, L.go A. Gemelli 1, 00168 Rome, Italy; 3 Department of Medicine and Health Sciences, University of Molise, Via Francesco De Santis, 86100 Campobasso, Italy

**Keywords:** Bone tumor, Curettage, Pathological fractures, Osteosynthesis in bone tumor, Bone grafts

## Abstract

**Background:**

The aim of the study was to evaluate whether the use of preventive osteosynthesis after curettage in benign and primitive low-grade malignant bone tumor localized in the distal femur in adult patients provides sufficient mechanical stability to the system as to allow weight-bearing and reduce the risk of postoperative fracture. Additionally, lower limb function after curettage and preventive osteosynthesis was evaluated.

**Materials and methods:**

We analyzed twelve cases of benign and low-grade malignant bone lesions of the distal femur in adult patients treated in our orthopedic department between 2008 and 2011 with curettage, bone filling and preventive osteosynthesis. All patients were treated with curettage with the use of high-speed cutters, plus liquid nitrogen as local adjuvant in low-grade malignant lesions, and filling of the lesion with bone graft or allograft or acrylic cement, followed by osteosynthesis.

**Results:**

No fractures or major complications were observed; good function of the knee was observed.

**Conclusion:**

We recommend preventive osteosynthesis after curettage in patients with very large lesions (>5 cm, >60 cm^3^) or high functional requirements, in obese patients, and when local adjuvants are used.

**Level of evidence:**

Level IV retrospective case-series study.

## Introduction

Curettage is widely used in musculoskeletal oncology to treat benign, or even aggressive lesions, some cartilaginous malignant lesions, and bone metastases [1].

The bone cavity resulting after curettage of the neoplastic lesion often requires the use of filling systems to ensure mechanical stability of the system, such as acrylic cement or bone grafts [2–4].

For most of the benign tumors, intralesional curettage and subsequent bone filling represents the treatment of choice, maintaining structural integrity and functional stability of the bone and adjacent joint. Depending on the tumor-specific risk of recurrence, adjuvant measures such as phenol instillation or cryotherapy with liquid nitrogen may additionally be applied due to their chemical and physical effects improving the local effect of curettage [1, 5–7].

**Fig. 1:**
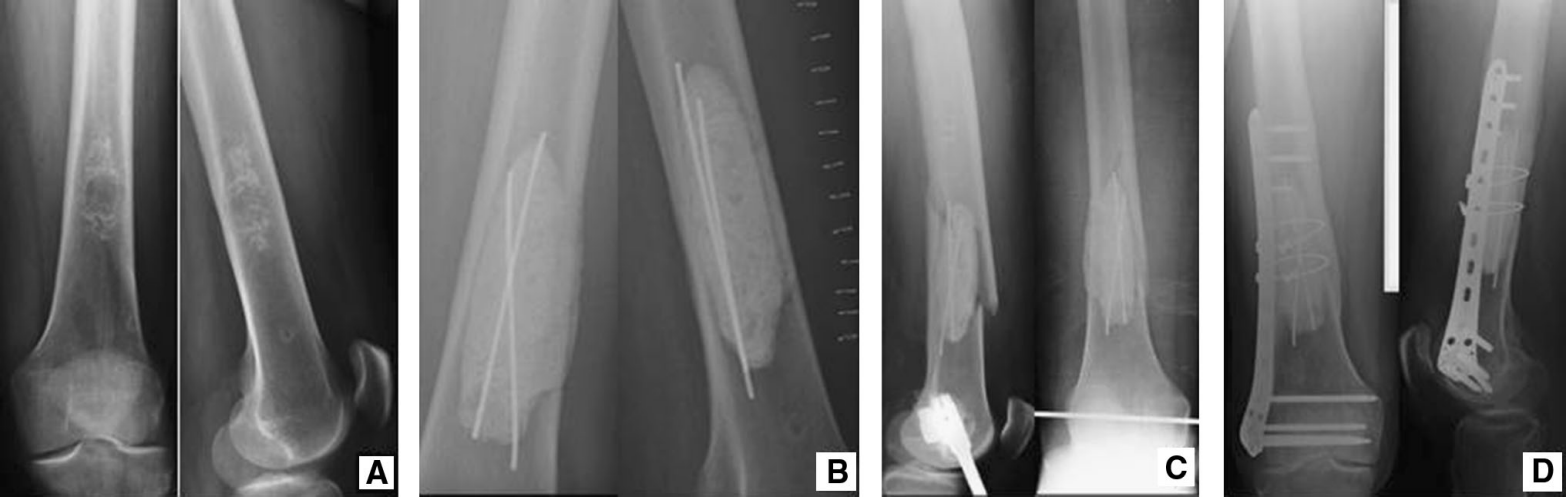
A low-grade chondrosarcoma of the femur (**a**) treated with curettage, filling and Kirschner wires (**b**). A fracture followed this procedure (**c**), treated through fixation with plate and locked screws (**d**)

However, these surgical techniques do not appear to be sufficient to ensure adequate mechanical stability in all cases, with consequent risk of fracture in some patients (Fig. 1).

The aim of the study was therefore to evaluate whether the use of preventive osteosynthesis after curettage in benign and primitive malignant bone tumors localized in the distal femur of adult patients provides sufficient mechanical stability to the system such as to allow load-bearing and reduce the risk of postoperative fracture. Additionally, lower limb function after curettage and preventive osteosynthesis was evaluated.

## Materials and methods

Between 2008 and 2011, twelve cases of benign and low-grade malignant bone lesions of the distal femur in adult patients were treated in our department with curettage, filling with bone or cement and preventive osteosynthesis.

Patients were enrolled retrospectively and a final follow-up was performed.

The mean age of patients (8 females and 4 males) was 31.6 ± 9.6 years (range 18–47 years).

All cases presented with unilateral lesions in the distal femur. The bone lesions of the patients with giant cell tumors and low-grade chondrosarcoma were filled with bone cement to take advantage of its adjuvant effect, while the remaining benign bone lesions were filled with allograft or bone graft. Adjuvant treatments were considered only in low-grade chondrosarcoma, giant cell tumors, chondroma and chondromixoid fibroma with liquid nitrogen (because absolute certainty of benignity of the lesion can only be assessed based on the entire specimen) to reduce the risk of local recurrence, improving the results of curettage alone [1, 4].

Demographics, clinical characteristics, histology and treatment of patients in our case series are described in Table 1.

**Table 1:** Demographics, clinical characteristics, histology and treatment

Case number	Age (years)	Gender	Clinical presentation	Dimension (cm)	Type of lesion	Treatment
1	18	M	Pain	7 × 3.5 × 3.5	Low-grade chondrosarcoma	Curettage, local adjuvant, filling with cement, osteosynthesis with LCP plate 4.5
2	25	M	Pain	10 × 4 × 3.5	Chondroma	Curettage, local adjuvant, filling with allograft, osteosynthesis with LISS plate
3	28	F	Pain	9 × 3.5 × 2.5	Giant cell tumor	Curettage, local adjuvant, filling with cement, osteosynthesis with LISS plate
4	45	F	Pain	8 × 3 × 3	Bone cyst	Curettage,filling with bone graft, osteosynthesis with LISS plate
5	44	F	Pain	8 × 3 × 2.5	Chondroma	Curettage, local adjuvant, filling allograft, osteosynthesis with LCP plate 4.5
6	33	F	Pain	12 × 3 × 3	Chondromixoid fibroma	Curettage, local adjuvant, filling with allograft, osteosynthesis with LCP plate 4.5
7	29	F	Pain	10 × 3.5 × 3	Chondroma	Curettage, local adjuvant, filling with allograft, osteosynthesis with LISS plate
8	31	M	Pain	11 × 4 × 3.5	Giant cell tumor	Curettage, local adjuvant, filling with cement, osteosynthesis with LISS plate
9	47	F	Pain limping	8 × 3 × 3.5	Low-grade chondrosarcoma	Curettage, local adjuvant, filling with cement, osteosynthesis with LISS plate
10	27	M	Pain limping	8 × 3.5 × 4	Chondroma	Curettage, local adjuvant, filling with allograft, osteosynthesis with LISS plate
11	17	F	Pain limping	7 × 3 × 3	Fibrous dysplasia	Curettage, filling with allograft, osteosynthesis with LCP plate 3.5
12	35	F	Pain	8 × 2.5 × 3	Fibrous dysplasia	Curettage, filling with bone graft, osteosynthesis with LCP plate 3.5

The plates used were the Less Invasive Stabilization System (LISS, Synthes) or the Locking Compression Plate 4.5 (Synthes).

Before treatment, all patients underwent comprehensive clinical and imaging assessment, including plain radiographs, computerized tomography (CT) and/or magnetic resonance imaging (MRI).

All patients were treated after surgery with a prophylactic dose of low molecular weight heparin, depending on weight and presence of risk factors for thrombofilia, for 5 weeks, and wore antithrombotic stockings. The following parameters were evaluated at follow-up: range of motion (ROM), complications, surgical revision, knee society scoring system (KSS).The clinical assessment of the patients through ROM and the KSS score was performed before and after the first surgery and after hardware removal (AHR). The hardware was removed in all patients after at least 1 year after surgery. The hardware was also removed in patients that had cement filling to reduce plate impingement, to try to obtain a better ROM, and because the patients were young (range 18–47 years).

Written informed consent was obtained from the patients for publication of this case series and any accompanying images. A copy of the written consent is available for review by the Editor of this journal.

Ethical approval was obtained from the Ethical Committee of the Catholic University of Sacred Heart in Rome.

## Results

**Fig. 2:**
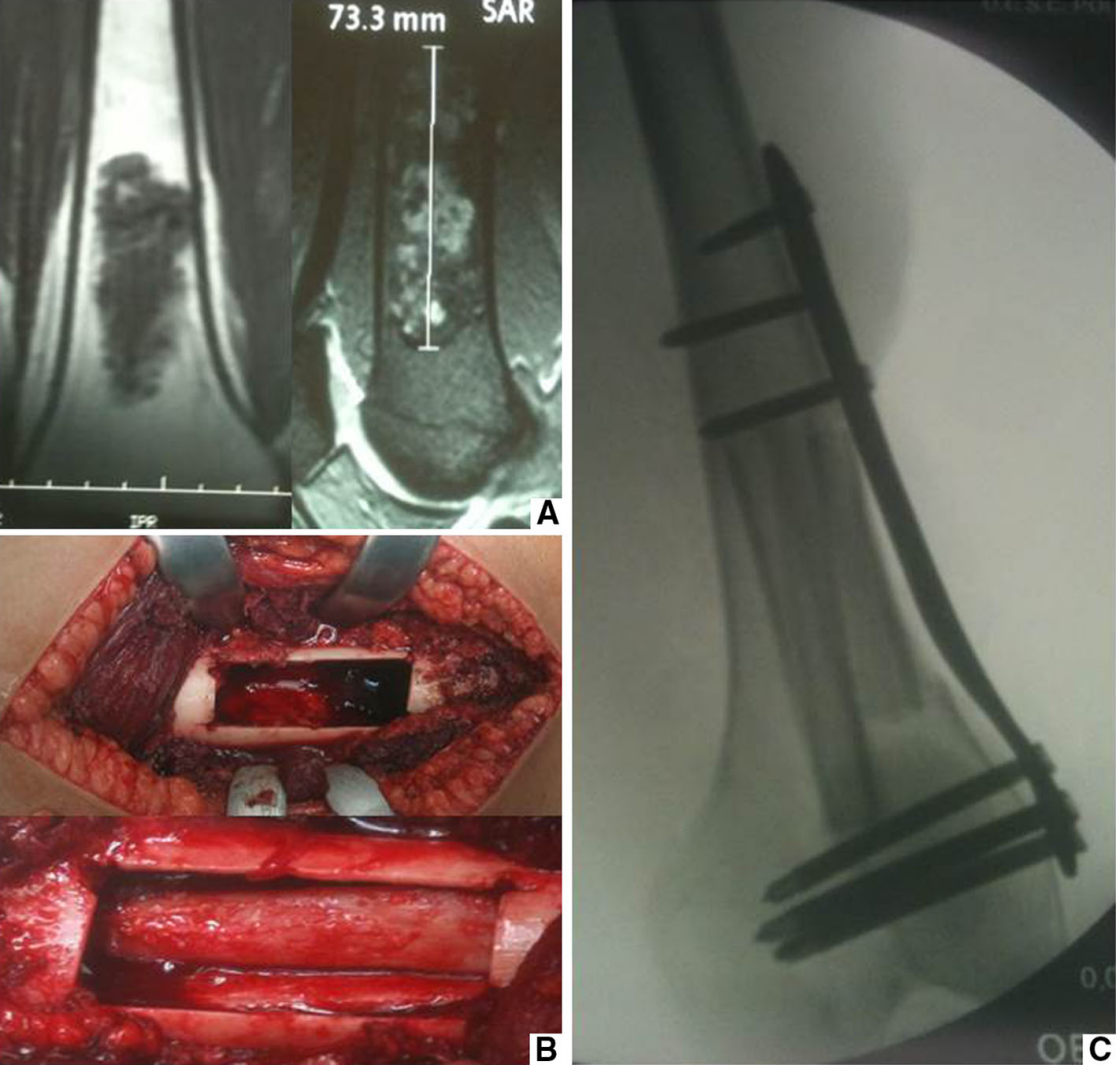
A low-grade chondrosarcoma of the distal femur (**a**), treated with curettage and filling with fibular allograft (**b**), and preventive osteosynthesis (**c**)

The surgical treatment consisted of curettage with the use of high-speed cutters, local adjuvant in selected cases and filling of the lesion with bone graft (2 cases), allograft (6 cases) and acrylic cement (4 cases), followed by fixation by plates with locked screws (Fig. 2).

All operations were performed by the same orthopedic surgeon.

No patient received chemotherapy or radiotherapy.

The minimum duration of follow-up was 36 months (range 36–72 months).

In the postoperative period, patients underwent early mobilization and continuous active and passive leg mobilization. Complete weight-bearing was proscribed for 1 month, subsequently progressive load was allowed with crutches, except for the patients treated with bone cement, who had a proscription for 20 days because of the better mechanical stability given by cement [1]. Active and passive knee mobilization was allowed immediately on the first postoperative day.

The functional and radiological outcomes are described in Table 2.

**Table 2:** Functional and radiological outcome

Case number	Complication		Alignment	Flexion operated	Flexion % contralateral	KSS (pre op)	KSS	Flexion operated (AHR)	Flexion % contralateral (AHR)	KSS (AHR)
1		None	Normal	140	100	Excellent	Excellent	140	100	Excellent
2		None	Normal	140	100	Excellent	Excellent	140	100	Excellent
3		None	Normal	140	100	Excellent	Excellent	140	100	Excellent
4		None	Normal	120	92	Excellent	Excellent	130	93	Excellent
5		Hardware pain	Normal	115	82	Good	Good	130	93	Excellent
6		None	Normal	120	86	Excellent	Good	135		Excellent
7		None	Normal	125	89	Excellent	Excellent	140	100	Excellent
8		None	Normal	120	86	Excellent	Good	130	93	Excellent
9		Superficial cutaneous infection	Normal	130	93	Excellent	Good	140	100	Good
10		None	Normal	140	100	Excellent	Excellent	140	100	Excellent
11		None	Normal	140	100	Excellent	Excellent	140	100	Excellent
12		None	Normal	130	93	Excellent	Excellent	140	100	Excellent

Before the first surgery, the KSS scores were excellent in 91.7 % and good in 8.3 %. After the first surgery (curettage plus osteosynthesis), the KSS scores were excellent in 66.7 % and good in 33.3 %. After the curettage, filling, and preventive osteosynthesis, the average ROM of the knee was 130.0° ± 9.8° with a percent of flexion than contralateral knee of 93.4° ± 6.6°. After hardware removal, an improvement was observed in the KSS (excellent in 91.7 %, good in 8.3 % of patients), in the average ROM of the knee (137.0° ± 4.5°) and in the percent of flexion compared with contralateral knee (98.1° ± 3.3°). Therefore, we observed a functional improvement after hardware removal although current literature fails to offer systematic guidelines and doesn’t support the routine removal of implants [8, 9].

No postoperative fractures in any patients were observed, either after the first surgery or after hardware removal.

No pain or limping was reported, except in the early postoperative time, usually solved with drugs (NSAIDs, paracetamol and codeine based drugs). In two cases patients had pain due to hardware that was solved after hardware removal. One case of cutaneous superficial infection was successfully solved with antibiotic therapy. No cases of cutaneous necrosis, delayed wound healing, deep venous thrombosis, infections or recurrence were observed.

## Discussion

Nowadays, in primary and secondary malignant bone tumors, hemiarthroplasty, arthroplasty and allograft-prosthesis-composite (APC), or curettage (associated with local adjuvants) followed by fixation with plate or intramedullary nail, represent the recommended techniques in most cases as they provide good mechanical stability, restore early limb function, and improve quality of life [10–13].

In benign and tumor-like lesions, isolated curettage has been curative in 95 % of cases; the recurrence rate is variable and depends on histotype of the lesion [14–16]. In order to reduce the risk of postoperative fracture favoring the process of bone consolidation [17], curettage has often been followed by filling with autologous bone grafts, allografts, polymethylmethacrylate (PMMA) or bone substitutes, to provide pain relief and early recovery, and to preserve structural integrity of the bone and adjacent joint [14, 18–24]. Nevertheless, some authors have reported that in some lesions (especially those in areas of load) this approach is not sufficient, since it may not assure mechanical stability of the system, with consequent risk of postoperative fracture [25]. Moreover, it has been reported that most benign bone defects will consolidate without the need for these bone substitutes [17, 22, 24].

Adequate exposure and accurate curettage are essential to maximize local control, regardless of whether adjuvants are used or not [15, 16]. The bone window must be large to allow an adequate curettage, especially in malignant lesions (to reduce the risk of recurrence). Therefore, this procedure often weakens the bone, with an increase of the torsional forces at the level of the bone window, making necessary the fixation with a plate.

There are few data in the literature that state the precise dimensions of the lesion that requires preventive osteosynthesis. Hirn et al. [17] showed a strong correlation between risk of postoperative fracture and both size and volume of the cyst. The average size of the cysts that fractured postoperatively was 108 cm^3^, and 58 cm^3^ for the cysts that did not fracture (*p* = 0.003). The risk of fracture was 5 % in patients with cysts less than 60 cm^3^, and 17 % for those with cysts larger than 60 cm^3^ (*p* = 0.01). The risk was 3 % when the maximum diameter of the cyst was ≤5 cm, but 15 % when the diameter was >5 cm (*p* = 0.02). Kundu et al. [26] also showed a correlation between risk of postoperative fracture and size, volume, and localization of the cyst. The risk of fracture was lower in long bones and pelvic bones with cysts less than 70 cm^3^, as compared to those with cysts larger than 70 cm^3^. They found that average size of the cysts that fractured in long bones was 126.52 cm^3^, and 49.352 cm^3^ for the cysts that did not fracture. The average size of the cysts that fractured in long bones of the lower limb was 142.11 cm^3^, and 53.094 cm^3^ for the cysts that did not fracture. There was no correlation between the risk of postoperative fracture and size/volume of the cyst in short bones.

Others authors concluded that in selected cases preventive osteosynthesis associated with filling with cement or bone grafts is recommended to reduce the risk of postoperative complications and provide early recovery of mobility [27].

In this case series, filling with bone graft (2 cases) or allograft (6 cases) or acrylic cement (4 cases) was always associated with preventive osteosynthesis of the lesion to increase the mechanical support of the final system.

PMMA is recommended by many authors [1, 4, 18] in aggressive benign lesions and low-grade malignant lesions. PMMA provides immediate stability and is always available in great quantity for large tumor cavities. Additionally, its exothermic reaction kills tumor cells, therefore decreasing the risk of recurrence. Allograft and bone substitutes are not generally indicated in small lesions [17].

The first limitation of this study was that the series was very small (only 12 patients), and the tumor histotypes different. Although these 12 patients represent a heterogeneous group of diagnoses (that would need different treatment options), we selected only lesions that needed a more aggressive approach due to the high risk of postoperative fractures: fibrous dysplasia with a very large lesion, giant cell tumor, chondrosarcoma grade I. Finally, the few cases of this series and the lack of a control group do not allow sufficient statistical power to support these conclusions: a larger and multicenter study should be considered in the future.

In conclusion, preventive osteosynthesis associated with curettage in benign and low-grade malignant primitive bone tumors of the distal femur in adult patients reduces the risk of postoperative fracture, and is associated with a good clinical and functional outcome. Preventive osteosynthesis could be indicated for very large lesions (>5 cm, >60 cm^3^) with residual large bone windows, lesions of the distal femur and loading zones, in obese patients and patients with high functional requests, when local adjuvants are used.
